# Sociobehavioral, Biological, and Health Characteristics of Riverside People in the Xingu Region, Pará, Brazil

**DOI:** 10.3390/ijerph20085542

**Published:** 2023-04-17

**Authors:** Dalberto Lucianelli Junior, Adenilson Leão Pereira, Ozélia Sousa Santos, Maria do Carmo Faria Paes, Yuji Magalhães Ikuta, Rodrigo Silveira, Fernanda Nogueira Valentin

**Affiliations:** 1 Postgraduate Program in Health of Amazon, Nucleus of Tropical Medicine, Federal University of Pará, Belém 66075-110, Brazil; 2 Faculty of Medicine, Airport Campus, University of Uberaba “Uniube”, Uberaba 38055-500, Brazil; 3 Faculty of Medicine, Federal University of Pará, Altamira 68372-040, Brazil; 4 Postgraduate Program in Biodiversity and Conservation, Federal University of Pará, Belém 66075-110, Brazil; 5 Institute for Environmental Research, Rheinisch-Westfälische Technische Hochschule Aachen University, 52074 Aachen, Germany; 6 Capital Campus: Cidade Universitária Armando de Salles Oliveira (CUASO), University of São Paulo, São Paulo 05508-065, Brazil

**Keywords:** education level, eating behavior, healthy lifestyle habits, non-communicable chronic diseases, machine learning

## Abstract

This study aimed to evaluate the sociodemographic, behavioral, and biological profile and its relationship with the emergence of chronic non-communicable diseases in riverside populations in the Xingu region, Pará, Brazil. Characteristics related to health indicators and which risk factors are considered most important were analyzed. This is a cross-sectional, exploratory, and descriptive study. The sample consisted of riverside people of over 18 years of both sexes. The sample size (n = 86) was calculated with a confidence level of 95% and a sample error of 5%. The K-means clustering algorithm was adopted through an unsupervised method to divide the groups, and the values were expressed as a median. For continuous and categorical data, the Mann-Whitney and chi-square tests were used, respectively, and the significance level was set at *p* < 5%. The multi-layer perceptron algorithm was applied to classify the degree of importance of each variable. Based on this information, the sample was divided into two groups: the group with low or no education, with bad habits and worse health conditions, and the group with opposite characteristics. The risk factors considered for cardiovascular diseases and diabetes in the groups were low education (*p* < 0.001), sedentary lifestyle (*p* < 0.01), smoking, alcoholism, body mass index (*p* < 0.05), and waist–hip ratio, with values above the expected being observed in both groups. The factors considered important so as to be considered to have good health condition or not were the educational and social conditions of these communities, and one part of the riverside population was considered healthier than the other.

## 1. Introduction

Environmental determinants may be related to lifestyle, and the onset of diseases can affect a given population’s health and quality of life. In addition, populations change their epidemiological profile due to the influence of chronic noncommunicable diseases (CNCDs) that are related to multiple causes [1].

According to the Global Burden of Disease Study, in Brazil, CNCDs, such as dyslipidemia, systemic arterial hypertension, and diabetes mellitus, correspond to about 75% of the causes of death [2], being a major problem for health services. Thus, the importance of surveillance of risk factors, such as sociodemographic, behavioral, and biological profiles, are effective ways to establish primary prevention measures and early detection of cardiovascular diseases [3]. Therefore, it is essential to adopt these practices, since it is still a challenge for managers and health professionals, especially in isolated areas where riverside populations live [4].

The main subsistence activities of these populations are based on artisanal fishing, family farming, and extractives [5]. However, these populations have characteristics that make it difficult for them to have access to the basic services that the urban populations have with a certain ease, since they live in rural and remote places, far from urban centers, have precarious financial conditions, precarious sanitary conditions, and difficult access to transportation, which makes it hard to go to hospitals and health clinics [6,7,8,9,10].

The riverside population of the middle Xingu River is distributed over several islands that are scattered along the river, making health care for this population difficult [9,11]. In addition, this is a complex region of conflicts and territorial and political disputes, marked by the installation of large enterprises, such as the Belo Monte Hydroelectric Power Plant (HPPBM), installed ten years ago, as well as gold mining and deforestation [9,12,13,14].

After the installation of HPPBM, the riverside population of this region, especially the families living in the middle Xingu River, suffered inestimable impacts, such as territorial expropriation (it is estimated that 56% of the population was relocated), contamination of the river (water contamination due to construction works and damming), and reduction of fishing resources, reducing the life quality and way of living of the remaining population [9,11,15,16].

Epidemiological information for the riverside populations of the middle Xingu region is incipient in Brazil, since the health services provided in these communities are sporadic, and the river population’s access to the urban area is limited due to financial conditions and difficulty of displacement [10]. In addition, factors such as low education, illiteracy, lack of basic sanitation, and poverty contribute to the emergence of endemic diseases that, until then, were unknown regarding their epidemiological profile within these populations [5,10].

Although Brazil has a single health system (Sistema Único de Saúde—SUS) where access is free to the population, it can be seen that, to some extent, it is exclusionary, because some vulnerable groups, such as the Amazon riverside people, are not efficiently served by such a system. This is due, in part, to geographic isolation and the deficiency of public policies aimed at these populations [17,18]. In addition, the demand for education in riverside communities faces many barriers, such as cultural, ideological, educational, and/or institutional [19]. This socioeconomic aspect associated with the lack of care can influence the absence of healthy habits in this population [20].

Although there are specific studies on the health profile of riverside populations [21], more in-depth and integrative studies are essential to better characterize the sociodemographic, behavioral, and biological patterns that contribute to the emergence of CNCDs, especially in the riverside population of the middle Xingu River, city of Altamira, state of Pará, Brazil.

The factors that influence CNCDs are complex and multifactorial, so the use of algorithms associated with machine learning, such as K-means, can identify non-linear patterns that allow a more accurate and possible classification association of seen lifestyle habits and the presence or absence of the most varied types of diseases [22,23,24]. Thus, the use of this algorithm with the input of data (e.g., sociodemographic, behavioral, and biological) obtained from the riverside populations of interest allows us to assess whether the behavior of these populations maintains a single general pattern or whether there are distinct patterns. In addition, it is possible to identify whether the variables that have not yet been studied in these populations are similar or not to other riverside communities based on the literature. Based on these assumptions, it is believed that the riverside population of the middle Xingu River has two riverine profiles: the first profile of people with less schooling, bad habits, and worse health conditions, as well as the second profile of people with opposite characteristics.

In this sense, this study evaluated in an exploratory way, through the machine learning technique, the sociodemographic, behavioral, and biological profile, as well as its relationship to the emergence of CNCDs in riverside populations in the middle Xingu region and analyzed the characteristics of these patterns regarding health indicators and which risk factors are considered most important.

## 2. Materials and Methods

### 2.1. Participants

**Figure 1:**
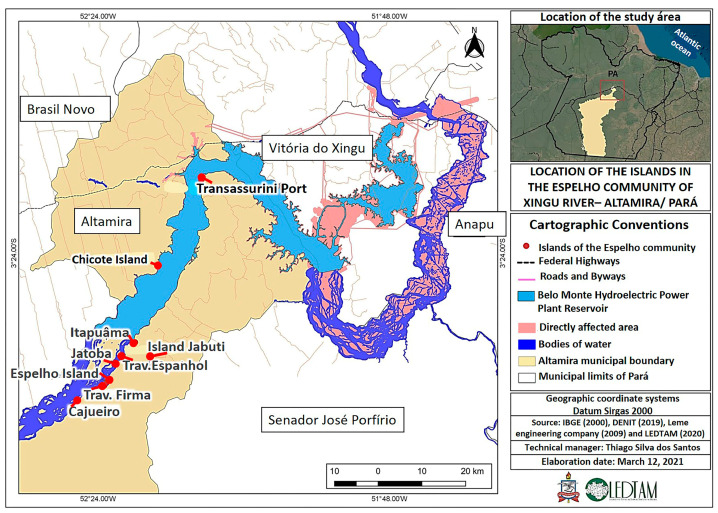
Location of the Espelho communities in the middle Xingu River.

This is a cross-sectional, exploratory, and descriptive study carried out from March to September of 2019 in a riverside community in the middle of Xingu River, Altamira town, Pará State. The study was developed in the Espelho community, consisting of ~60 families, distributed in small communities (Cajueiro, Chicote, Espelho, Jabuti, Itapuâma, Jabota, Transassurini, Espanhol, and Firma) along the banks of the middle Xingu River (Figure 1). Two expeditions were carried out that took place in the Chicote Island community, considered a geographically privileged place among the other communities, so it was used as a data collection base. Regarding the profile of the interviewees, the inclusion criterion was for convenience, so the profile was traced by the presence of riverine people in each field campaign, which was widely publicized a month in advance.

The sample size resulted from the total number of riverine people present in each field survey. Thus, 40 families were accepted to be part of this research, which had an average of 4.3 ± 1.59 people per family, totaling 172 individuals. Within this group, an average of 2.2 ± 0.79 people per family were over 18 years old, totaling 86 individuals of both sexes who signed informed consent and answered the questionnaire.

People with cognitive problems and those who refused to participate in the research at any time of the action, even if they signed the consent form, were excluded.

To assess the representativeness of the number of individuals who agreed to participate in the research, Slovin’s formula was used: n = N/(1 + Ne2), where n = number of samples, N = total population, and e = error tolerance (level) [25,26], based on a statistical power of 90%. After applying the equation, it was verified that this study would need at least 84 individuals. Therefore, the sample obtained is representative. Furthermore, the sampling effort of this study is similar to that of others that analyzed small riverside communities [27,28,29].

This study was approved by the Ethics Committee of the Tropical Medicine Center at the Federal University of Pará (Opinion No. 3678493), in compliance with Resolutions 441/2011/CNS e 466/2012/MS.

### 2.2. Procedures

**Figure 2:**
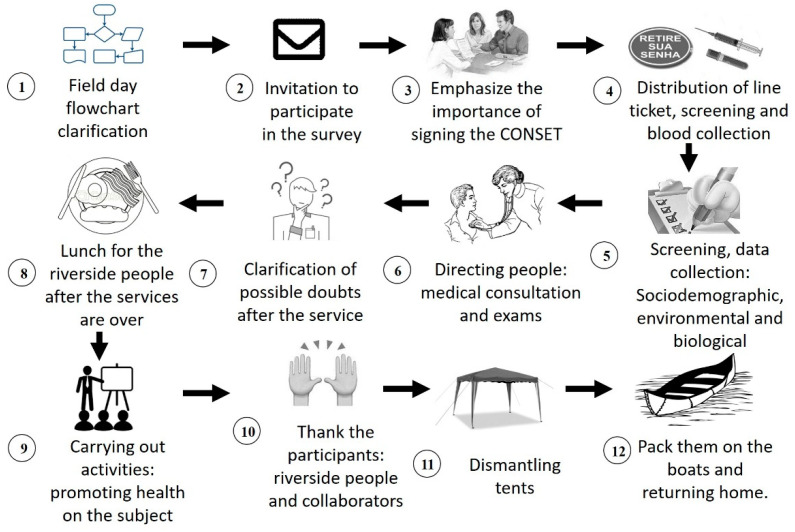
Flowchart of service on the day of the action.

In Figure 2, it is possible to observe the chronological flow and the operational procedures developed during the research. We emphasize that consent was read and explained to all participants who could neither read nor write, who, after agreeing, put their fingerprints on the document. Data collection from the participants included: (1) sociodemographic characteristics: sex, ethnicity, reading, and education level; (2) behavioral habits: smoking, classification of smokers (light or moderate to heavy categories) [30], alcohol consumption, physical exercise, healthy diet (including weekly consumption of fish, nuts, fruits, vegetables)/bad diet (fried foods, soft drinks, sausages, among others); (3) biological predictors: age, blood pressure, body mass index (BMI), waist/hip ratio (WHR), and personal physiological history (dyslipidemia, arterial hypertension—SAH, diabetes—DM, stroke—CVA, cardiovascular disease—CVD).

Blood samples were taken to evaluate the lipid profile: total cholesterol (TC), HDL, LDL, triglycerides (TG), and blood glucose, following established protocols [31,32].

### 2.3. Data Analysis

Assuming that the development of CNCDs is multifactorial [1], which means that it can arise through different factors (e.g., level of physical exercise, eating habits, smoking), the sample was divided by clustering, using the algorithm K-means clustering method that, through an unsupervised method, learns how all the variables of a data set are related to each other by dividing the participants into different predetermined groups, which can help to discover different hidden patterns present in the data. This grouping occurs by minimizing the sum of squares of the distances between the data and corresponds to the geometric center of a characteristic, called the centroid [33].

Based on the hypothesis of the existence of an association between social factors, health, and habits that have an impact on health, we suggest that there could be two profiles of people: the profile of low education or no studies, with bad health habits and worse health conditions; and the profile with opposite characteristics. Therefore, the K-means clustering algorithm was used to cluster the sample into two main groups [34]. 

For the construction of the model, 28 variables were used (two sociodemographic variables, 17 biological variables, and nine behavioral variables). Orange Data Mining 2.27 software was used to determine the groups (Supplementary Materials).

We highlight that only two sociodemographic variables were inserted because there are biological variables (sex, age, and ethnicity) already evidenced in the literature as factors associated with a sociodemographic level, so they were considered in the biological variables and not in the sociodemographic variable [4,35,36,37]. The variable “fish consumption” was emphasized, since this food is rich in omega-3 fatty acid, which is a nutrient known to be associated with the prevention and protection of cardiovascular diseases [38]. On the other hand, the consumption of fish by the population studied may be a risk factor for mercury contamination, since it has been reported that many species of fish in the Xingu River have levels of methylmercury concentration (the organic form of the metal heavy mercury) far above what is tolerable by the human body and which has a high power of bioaccumulation along the food chain [39,40,41,42].

### 2.4. Statistical Analysis

Group characteristics were expressed as a median and interquartile range, mean, standard deviation, and proportions. The Mann-Whitney and chi-square tests were used for comparisons between groups, with Fisher and Yates corrections, and a *p*-value < 0.05 was adopted as significant. Residual adjustment >2 was adopted for significant categorical analyses.

The effect size was considered to support the importance of differences between groups. For continuous data, the interpretations followed the Cohen table (2013). For categorical data, effect sizes were observed through φ in 2 × 2 tables, assuming “Null effect” for φ < 0.10, “Small effect” for φ < 0.30, “Moderate effect” for φ < 0.50, and “Large effect” for higher values. In tables > 2 × 2, the sizes were observed by Cramer’s V, whose interpretations of null, small, moderate, and large effects were performed, respecting the variations according to the increase in degrees of freedom [43,44].

The algorithm multilayer perceptron [45] was used to assess and classify the degree of importance of each variable in determining the groups. It is a supervised machine learning algorithm that, through artificial neural networks, can find non-linear patterns among different variables in a dataset and in response provides a prediction of some predetermined variable.

Only significant variables (*p* < 0.05) were inserted in the input layer for the prediction of groups by K-means, of which four were biological, four were behavioral, and one was sociodemographic. Numerical variables were rescaled at intervals between 0 and 1. The sample was randomly divided into two data sets, with 70.2% of the sample (n = 60 individuals) used to train the algorithm and 29.8% used for the test (n = 26 individuals). The architecture of the algorithm was automatically determined by the software, and the most adequate pattern to structure the neural network was the use of a neuron and a hidden layer. The activation functions used in the hidden layer and output layer were tangent hyperbolic and softmax, respectively. To minimize a possible effect of overfitting (a false response in the network), the algorithm was applied three times, and the application chosen was the one with the lowest cross-entropy error, 0.026 (training sample) and 0.013 (test sample).

To calculate the importance of each variable in dividing the groups, a sensitivity analysis was performed based on the combined training and test samples, creating a table displaying each variable’s importance rating.

IBM Statistical Package for the Social Sciences (SPSS) 23.0 software was used for the evaluation procedures of the division of the groups.

## 3. Results

After dividing the sample composed of 86, the K-means algorithm defined two groups: group 1 composed of 47 people with major socio-demographic vulnerability, poor health habits, and worse health conditions, and group 2 composed of 39 people with profiles of opposite characteristics.

Regarding the sociodemographic descriptors (Table 1), when the participants were asked about knowing how to read or not, 100% of the people in group 1 said they did not know how to read, while group 2 was the opposite, revealing approximately half of this population. As for education, 59% of people who declared they had not studied belonged to group 1. In addition, 16 people declared having attended elementary school. However, they could not read. Among the people declared to be literate and who had attended elementary school (46.8%) or high school (10.6%), they were in group 2. Only one person declared having graduated. However, this did not influence the groups among the riverside people. The effect size was highly significant (*p* < 0.001) for reading and schooling, evidencing a low education profile or without studies. The other social criteria were not relevant between the groups (*p* > 0.05).

Regarding the behavioral descriptors, it was observed that, in group 1, people smoked for a longer period (0–15 years; Table 2). Characteristics, such as the number of cigarettes smoked per day (Table 2) and the rate of smokers and former smokers (Table 3), did not influence the comparison between the groups. It is important to note that riverside dwellers who reported being smokers (7% of the population) were included in the “light” (≤10 cigarettes/day) or “moderate to heavy” (>10 cigarettes/day) categories.

As for the frequency of alcohol consumption (Table 2), people in group 2 consume alcohol more frequently every week when compared to group 1 (*p* < 0.05). However, of the people who consume alcoholic beverages, 42.6% are in group 2, representing double those belonging to group 1 (Table 3).

A percentage of 39.5% of the riverine people declared to practice physical exercises. The weekly frequency of physical exercise was lower in group 1 (0.0 times) than in group 2 (1.5 times) (*p* < 0.01; Table 2).

Regarding the consumption of fish per week (Table 2), it was observed that they consume fish three to four times a week and that this frequency did not differ between the groups (*p* > 0.05). There was no difference between the groups as to whether or not they had a healthy diet (Table 3). However, most riverside dwellers stated that they preferred a healthy diet, with 61.5% in group 1 and 70.2% in group 2 (Table 3).

Considering the lipid profile, even if the values are within the acceptable limit [46], the riverside people in group 2 tended to have lower values of total cholesterol and LDL when compared to group 1 (*p* < 0.05; Table 2). The other biochemical tests, blood pressure measurement, WHR, and physiological background (SAH, CVA, CVD) showed no differences between the groups (*p* > 0.05). In addition, SAH was within the normal range. However, it was observed that the systolic blood pressure (SBP) was at the limit (SBP = 130 mmHg) recommended by the Brazilian Society of Cardiology [46].

Although the WHR did not differ between the two groups, both presented values above the desired level (Table 2). Men had WHR ≥ 0.90 cm, and women had values ≥ 0.85 cm. The BMI of this population differed between the groups, where group 1 had higher values when compared to group 2 (*p* < 0.05; Table 2). However, both groups were considered pre-obese according to the WHO [47]. Although SAH and DM did not differ between the groups, these variables had an average frequency of the population, in general, of 21.3%, including those who reported SAH, and 4.7% reported having DM and SAH. 

The biological descriptors observed in group 1 showed people of more advanced ages when compared to group 2, with a mean age of 55 years (49–62 years) (*p* < 0.001; Table 2). Although age was significant, the effect size was small.

**Figure 3:**
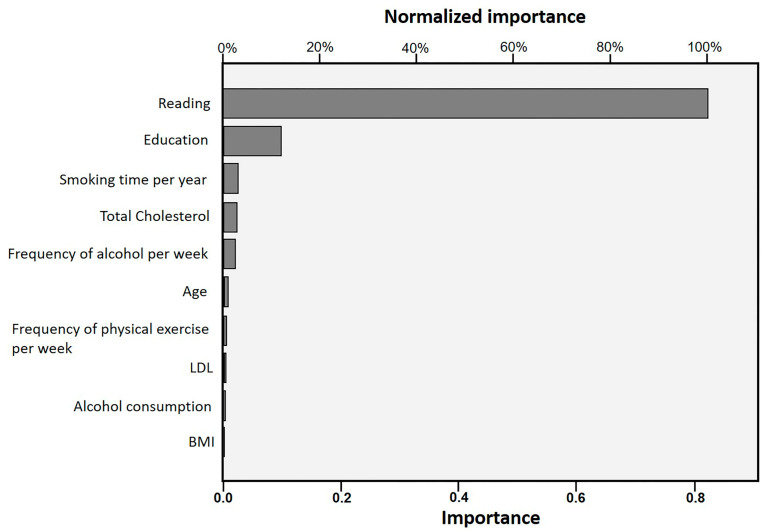
Ranking of the importance of quantitative variables rescaled in intervals between 0 and 1, showing the degree of importance of the variables (from highest to lowest). Personal source.

Thus, it was evidenced by multilayer perceptron that, of the most important variables, social factors, such as reading (100%) and level of education (11.8%), were the factors that most influenced this study; then, the second most important were behavioral descriptors, such as smoking time (2.7%), total cholesterol (2.6%), alcohol frequency (2.3%), and others. Although age had an influence, the effect size was small, and it has a lower importance (0.9%) than the other variables, as shown in Figure 3.

## 4. Discussion

This study evaluated the relationship of sociodemographic, behavioral, and biological patterns in an Amazonian riverine population of the middle Xingu River to identify the factors that most influence one’s health profile. We assume that this population has two profiles, and our analyses show that there are two distinct groups: a group with low education and poor health, and another group with opposite characteristics. In addition, we found that the educational and social conditions together were the most relevant aspect for having good health conditions in this population.

We observed that all people in group 1 are illiterate, regardless of having attended elementary school or not, showing that the lack of reading may lead people to be unable to read or interpret, for example, a medication leaflet, tables of nutritional information present in processed and industrialized foods, and documents that may provide information on health education. The population’s level of schooling was low, evidenced mainly in group 1, with 59.9% of the riverside people without education, and although the other group has a better educational index, most have only elementary school. These data are particularly important, since they agree with other studies carried out in the Amazon and in other regions of the world, where a low level of schooling is a risk factor that can influence the development of CNCDs and other diseases [27,48,49,50,51].

In this context, we believe that low education can make it difficult or impossible for the population to understand the importance of healthy lifestyles, such as a good diet, physical activity, and reduced consumption of industrialized products. Recently, [52] found that the level of education can influence the dietary profile of the population of London, United Kingdom, in three ways: (i) low education is linked to diets rich in carbohydrates and low in fiber; and (ii) low education is also associated with higher consumption of sweets and red meat, and high education is associated with higher consumption of fruits, vegetables, and fish. In Latin America, it was observed that the main factors that hinder the state of health conditions in this population are: socioeconomic inequality (e.g., low education), social/geographical isolation and cultural barriers, and linguistic and political, which mainly affect indigenous and other populations living in rural areas [53]. Considering Brazilian Amazon populations, studies in riverside populations of the Pará (Tapajós River and Tucuruí Lake) and Amazonas (Negro River and Solimões River) states demonstrate that the profile or status of educational and bad habits (food and health) of riverine people are risk factors for the development of the metabolic syndrome and cardiometabolic diseases [21,27,48,54,55].

Regarding the smoking habit, the variable “time of smoking” was the most influenced in the study, especially in group 1, which was also considered to have poor health conditions. In riverside communities in the Amazon, smoking was associated with weight gain, the development of DM2, the onset of systemic arterial hypertension, metabolic syndrome, and chronic kidney disease [56,57]. Although the harmful effects of smoking on the body and the emergence of diseases are already widely known [58,59], the data reported here are particularly important, since they involve traditional populations that are historically neglected or excluded by public health policies in Brazil.

Considering alcohol consumption, the results showed that the consumption of alcoholic beverages and educational qualifications (literate) belonged to group 2, corroborating previous studies that associate the existence of a higher consumption of alcohol among people with better education [60,61,62,63]. Regarding the consumption of alcohol in riverside communities, it was reported that, in riverside communities of Amazonas and Paraíba states, it reaches 34.8% and 30.4%, respectively [56,64]. These studies corroborate our data, which pointed out that about 30% of riverside people are considered consumers of alcoholic beverages. The level of education shapes social position and opportunities in life, promoting circumstances that favor alcohol consumption [65]. 

Contrary to previous studies on dyslipidemia in riverine and quilombola populations in the region [4,66], this study showed that the values for cholesterol and LDL were considered desirable for both groups of this population. However, group 2 presented relatively lower values, suggesting that a part of the population has healthier lifestyle habits than the other. Perhaps the limitations to access to processed foods, due to poverty and low financial resources, are an influencing factor [67,68,69].

The effect size of age, despite being significant, was small concerning other sociodemographic and biological variables, such as education and lipid profile, respectively, thus not influencing the health of the riverine [70]. On the other hand, the level of knowledge and age may have a possible association, since the riverside people who had no education were in the same group as those who were older (group 1), as also demonstrated by Peres [71]. This may be mainly associated with the absence of educational programs in the past decades, as well as financial difficulties and difficulties with locomotion and transportation [19].

The population studied presented a typical food profile of riverside populations in the Amazon region, in which riverine people reported having a good diet (natural food) based on vegetables, Brazil nuts, fruits, and mainly fish, the latter item being consumed three to four times per week [55,72]. We highlight the “fish consumption” factor for two interesting aspects: (i) a positive aspect, since there is a high concentration of unsaturated fats and omega-3 fatty acids in this food, which can substantially contribute to the prevention of cardiovascular diseases in this population, since this nutrient has been proven to prevent this disease [38,73]; and (ii) a negative aspect, since there is a strong possibility that this food is contaminated by methylmercury, which in turn can bioaccumulate in riverine people who consume them [39,40,41,42]. Indeed, Souza-Araujo and collaborators (2022) [42] identified fish contamination and mercury biomagnification in food webs in the Belo Monte Hydroelectric Power Plant, in the middle of Xingu River, Pará state. Recent studies have reported mercury contamination of traditional (e.g., riverine and indigenous) and urban communities in the lower Tapajós basin and in the Tucurí Lake influence area, Pará state [40,41,74]. Therefore, it is possible that the population studied may be at risk of methylmercury contamination, since they may be consuming contaminated fish.

We observed that a large part of the population studied had blood pressure within the limits of what is recommended by the Brazilian Cardiology Medical Society [46]. This condition may be associated with possible mercury exposure, since concentrations of this metal in the bloodstream are positively associated with blood pressure, hypertension, and other cardiovascular diseases. In addition, the exposure dose is an important factor in determining the effects on hypertension [75,76,77].

Despite these results, the riverside population of the current survey reported having some of the CNCDs, such as SAH and DM. Even though they do not influence this study, these variables presented an average frequency of the general population of 21.3% reported to have SAH and 4.7% declared to have DM and SAH. Studies carried out in riverside communities in the interior of Amazonas show a higher prevalence of SAH (30.7%) and DM (8.9%), whereas, in the interior of Pará, 20.47% reported being hypertensive, and 4.13% reported having diabetes, and such prevalence corroborates with our studies [56,78].

Regarding the frequency of physical exercises per week, a low frequency of exercise was evidenced in both groups, and less than half of the population is a practitioner. Such results confront the idea that populations residing in rural areas have higher levels of physical activity, which is most often represented by walking [79,80,81].

Anthropometric indicators showed that BMI and WHR presented values above the expected in both groups evaluated, as well as in both sexes, considered pre-obese for BMI and high risk for metabolic complications for WHR, suggesting an excess of intra-abdominal adipose tissue, as also evidenced by other studies [47,82,83]. According to Pereira et al. [84], the WHR has a greater predictive capacity for hypertension, allowing greater discrimination of people at risk of chronic diseases. However, BMI and WHR were not determining predictors among the risk factors for classifying the importance of quantitative variables in the present study, thus not indicating a possible association with CNCDs.

In this sense, public policies should be worked on with these riverside populations, which lack medical care. The study carried out by Machado et al. [20], with riverside populations of the lower Madeira in Rondônia, used telemedicine as a technological resource for health promotion and prevention and developing the population’s responsibility for a better quality of life in the region.

The Brazilian educational system does not have public policies of national scope aimed specifically at the traditional populations (e.g., indigenous, riverine, quilombolas, etc.), so the only program that exists does not meet the breadth of distribution of these populations in the national territory. For example, the government of the state of Pará launched the program “Modular Education Organization”, which is regulated by law N°. 7806 of 29 April 2014, and that aims to bring education and literacy to traditional populations and the interior of the state. However, although this program has significantly contributed to the schooling of approximately 36,000 students from 465 locations in the interior of the state [85], it is still limited and cannot serve more distant and isolated communities, such as the population of the region around the middle Xingu River, so this population remains unassisted by public education policies. Therefore, given the importance of education for the critical understanding of people regarding their social context, robust public policies for schooling and health education (in cooperation with states, municipalities, and the federal government) are essential to mitigate the impact of this factor on the quality of life and health of traditional populations.

Overall, this study could confirm the hypotheses based on the literature, that a person with no or less education is unlikely to have knowledge and information about how harmful certain behaviors are. Therefore, they will not change them and, as a result, will have worse health indicators. Only alcohol intake showed a different pattern from the other factors, since its influence could be observed within the best level of education, probably because education provides better social conditions and opportunities, favoring this consumption. Finally, healthy eating, mainly due to the high consumption of fish, was considered a positive criterion for the health of riverside dwellers.

The riverside population studied has an important peculiarity, which is its relative geographic isolation, since this population is surrounded by numerous waterfalls in the middle Xingu River, which makes navigation in this region difficult. In addition, with the installation of the Belo Monte Hydroelectric Power Plant, many families lost their land and were “relocated” to another region, so that the local riverine population was reduced. Therefore, the difficult access to the collection site, the high financial cost of the study, and the population size profile of the studied community (considered small and dispersed along the middle Xingu River) may have hindered the adherence and participation of a greater number of riverine people in the survey. However, we believe that the sampling effort did not affect our results, which are in agreement with other studies that analyzed small or large Amazonian riverside communities. 

Finally, although this study brings relevant results, especially regarding the degree of importance of health indicators, the research had some limitations, such as the experimental design, which has a transversal characteristic. Studies that are more longitudinal are needed to better understand how the population would behave if there was an intervention and monitoring by health professionals about the most important variable: education. Could it be that by encouraging and intervening in education, positively, the other indicators would also improve? Another limitation was that the procedure was not conducted in a laboratory, and the process was not systematized and controlled. Despite this, the study valued a view of the subject in a real environment. 

## 5. Conclusions

There are two patterns in the riverside community, being a pattern of people with low education, with longer exposure to cigarettes and low frequency of physical exercise, factors that can be considered as risk factors for cardiovascular diseases and diabetes in this population and consequently can affect your health. Additionally, the other pattern has opposite characteristics, being considered healthier. On the other hand, food, mainly due to the high consumption of fish, was considered a positive criterion for the health of riverside dwellers in both groups. 

This is the first study of this population, which has important peculiarities that distinguish it from other Amazonian riverine populations. Finally, the most important variables for determining these groups were sociodemographic (education), behavioral (smoking and weekly alcohol consumption), and health (cholesterol).

## Figures and Tables

**Table 1:** Categorical data on the sociodemographic profile of the riverside population of Group 1 and Group 2 in the Xingu River region.

Descriptors	Group 1 n = 39	Group 2 n = 47	Effect Size	*p*
***Sociodemographic***				
**Sex**	F = 51.3%	F = 48.9%	Φ = 0.02	0.83
	M = 48.7%	M = 51.1%		
**Ethnicity**				
Asian	2.6%	4.3%	*V* = 0.23 ^++^	0.35
White	17.9%	21.3%		
Indigenous	2.6%	6.4%		
Mixed race	43.6%	53.2%		
Black	33.3%	14.9%		
**Education**				
No education	59.0% ^a^	0.0% ^a^	*V* = 0.77 ^+++^	< 0.001 ***
Initial Elementary School	41.0%	40.5%		
Final Elementary School	0.0% ^b^	46.8% ^b^		
High School	0.0% ^c^	10.6% ^c^		
University graduate	0.0%	2.1%		
**Reading**				
No	100.0%	0.0%	Φ = 1.00 ^+++^	< 0.001 ***
Yes	0.0%	100.0%		

The letter “a” represents the highest adjusted residual value (above 2) and the subsequent letters characterize lower values, respectively, representing the categories that influenced the statistical significance (*p*-value < 0.05) between the groups. *** *p* < 0.001. ^++^ moderate effect, ^+++^ Large effect.

**Table 2:** Continuous data on the behavioral and biological profile of the riverine population of Group 1 and Group 2 in the Xingu River region.

Descriptors	Group 1 n = 39	Group 2 n = 47	Effect Size	*p*
***Behavioral***				
Smoking Time/year	0.0 (0–15)	0.0 (0–0)	0.04	<0.05 *
Number of cigarettes/day	0.0 (0–0)	0.0 (0–0)	0.00	0.50
Frequency Alcohol/week	0.0 (0–0)	0.0 (0–3)	0.04	<0.05 *
Exercise Frequency	0.0 (0–0)	1.5 (0–4)	0.11 ^+^	<0.01 **
Fish Consumption/week	3.0 (2–6)	4.0 (2–6)	0.01	0.32
***Biological***				
SBP	130.0 (120–142)	130.0 (120–140)	0.01	0.30
DBP	80 (80–90)	80 (80–92)	0.00	0.80
WHR	1.0 (0.9–1.0)	0.9 (0.9–1.0)	0.01	0.27
BMI kg/m^2^	29.4 (25–34)	27 (24–30)	0.05	<0.05 *
Total Cholesterol mg/dL	185 (165–215)	166 (140–200)	0.07	<0.05 *
HDL mg/dL	55 (50–68)	54 (41–65)	0.02	0.23
LDL mg/dL	103 (81–123)	83.8 (72–104)	0.08	<0.05 *
Triglycerides mg/dL	120 (85–159)	115 (80–183)	0.00	0.90
Blood glucose mg/dL	70 (68–85)	72 (70–84)	0.01	0.40
Age	55.0 (49–62)	40.0 (32–48)	0.29 ^+^	<0.001 ***

Continuous data influenced the statistical significance (*p*-value < 0.05) between the groups. * *p* < 0.05, ** *p* < 0.01, *** *p* < 0.001. ^+^ Small effect. Abbreviations: SBP: systolic blood pressure; DBP: diastolic blood pressure; WHR: waist–hip ratio; BMI: body mass index; HDL: high-density lipoprotein; LDL: low-density lipoprotein.

**Table 3:** Categorical data on the behavioral and biological profile of riverine populations in Group 1 and Group 2 in the Xingu River region.

Descriptors	Group 1 n = 39	Group 2 n = 47	Effect Size	*p*
***Behavioral***				
**Smoker**				
No	89.7%	95.7%	Φ = 0.12 ^+^	0.40
Yes	10.3%	4.3%		
**Ex-smoker**				
No	64.1%	85.1%	*V* = 0.24 ^++^	0.09
Yes	25.6%	10.6%		
No reply	10.3%	4.3%		
**Alcohol consumption**				
No	79.5%	57.4%	Φ = 0.23 ^+^	<0.05 *
Yes	20.5%	42.6%		
**Healthy eating**				
No	38.5%	29.8%	Φ = 0.09	0.40
Yes	61.5%	70.2%		
***BIOLOGICAL***				
**SAH**				
No	74.4%	83.3%	Φ = 0.10 ^+^	0.33
Yes	25.6%	17.0%		
**Diabetes**				
No	94.9%	95.7%	Φ = 0.02	1.00
Yes	5.1%	4.3%		
**Stroke**				
No	100%	97.9%	Φ = 0.10 ^+^	1.00
Yes	0.0%	2.1%		
**CVD**				
No	84.6%	85.1%	*V* = 0.10 ^+^	0.87
Don’t know	15.4%	12.8%		
Other	0.0%	2.1%		

Categories that influenced the statistical significance (*p*-value < 0.05) between the groups. * *p* < 0.05. ^+^ Small effect, ^++^ moderate effect.

## Data Availability

Data is contained within the article or Supplementary Materials.

## Supplementary Materials

The following supporting information can be downloaded at: https://www.mdpi.com/article/10.3390/ijerph20085542/s1, Table S1: List of variables used in the construction of the clustering model by the K-means algorithm.

## References

[1] Brasil M.S. (2013). Guidelines for the Care of People with Chronic Diseases in Health Care Networks and in Priority Lines of Care.

[2] GBD (2016). Risk Factors Collaborators. Global, regional, and national comparative risk assessment of 79 behavioural, environmental and occupational, and metabolic risks or clusters of risks, 1990–2015: A systematic analysis for the Global Burden of Disease Study 2015. Lancet.

[3] Singhal A. (2014). The global epidemic of noncommunicable disease: The role of early-life factors. J. Int. Nutr. Achiev. Millenn. Goals Beyond.

[4] Oliveira B.F.A., Mourão D.S., Gomes N., Costa J.M.C., Souza A.V., Bastos W.R., Fonseca M.F., Mariani C.F., Abbad G., Hacon S.S. (2013). Prevalence of arterial hypertension in riverside communities on the Madeira River, Western Brazilian Amazon. J. Cad. De Saude Publica.

[5] Hacon S.S., Dórea J.G., Fonseca M.F., Oliveira B.A., Mourão D.S., Ruiz C., Gonçalves R.A., Mariani C.F., Bastos W.R. (2014). The influence of changes in lifestyle and mercury exposure in riverine populations of the Madeira River (Amazon Basin) near a hydroelectric project. Int. J. Environ. Res. Public Health.

[6] Arrifano G.P.F., Martin-Doimeadios R.D.C.R., Jiménez-Moreno M., Augusto-Oliveira M., Souza-Monteiro J.R., Paraense R., Machado C.R., Farina M., Macchi B., Do Nascimento J.L.M. (2018). Assessing mercury intoxication in isolated/remote populations: Increased S100B mRNA in blood in exposed riverine inhabitants of the Amazon. J. Neurotoxicol..

[7] Franco E.C., Santo C.E., Arakawa A.M., Xavier A., França M.d.L., De Oliveira A.N., Machado M.A.M.P., Bastos R.S., Bastos J.R.M., Caldana M.L. (2015). Health promotion on amazonic riverside population: Experience report. J. Rev. CEFAC.

[8] Gama A.S.M., Fernandes T.G., Parente R.C.P., Secoli S.R. (2018). Health survey in riverside communities in Amazonas, Brazil. J. Cad. De Saúde Pública.

[9] Magalhães S.B., Cunha M.C. (2017). Study on the Compulsory Displacement of Riverside Dwellers in Belo Monte: SBPC Report.

[10] Santos Sousa I., Sousa F.C., Sousa R.M.S. (2009). Living condition and water and sanitary situation in communities in the sphere of incluence of the gas pipeline Coari-Manaus in Macacapuru, state of Amazon, Brazil. J. Hygeia-Rev. Bras. De Geogr. Médica E Da Saúde.

[11] Weißermel S. (2020). Towards a conceptual understanding of dispossession—Belo Monte and the precarization of the riverine people. J. Novos Cad. NAEA.

[12] Gonçalves A.C.O., Cornetta A., Alves F., Barbosa L.J.G., Alves F. (2016). Belém and Abaetetuba. The Socio-Environmental Function of the Union’s Heritage in the Amazon.

[13] Lucas E.W.M., De Sousa F.A.S., Dos Santos F.D.S., Rocha-Júnior R.L., Pinto D.D.C., Da Silva V.P.R. (2021). Trends in climate extreme indices assessed in the Xingu river basin-Brazilian Amazon. J. Weather Clim. Extrem..

[14] Siqueira J.M., Dal’Asta A.P., Amaral S., Escada M.I.S., Monteiro A.M.V. (2017). The Middle and Lower Xingu: The response to the crystallization of different temporalities in the production of regional space. J. Rev. Bras. De Estud. Urbanos E Reg..

[15] De Francesco A., Carneirom C. (2015). Atlas of the Impacts of HPP Belo Monte on Fisheries.

[16] Silveira M. (2016). The Implementation of Hydroelectric Plants in the Brazilian Amazon, Socio-Environmental and Health Impacts with the Transformations in the Territory: The Case of the Belo Monte HPP. Ph.D. Thesis.

[17] Brasil M.S. (2015). The National Policy for the Comprehensive Health of Rural, Forest and Water Populations and the Environment.

[18] Pontes F.A.R., Silva S.S.d.C., Bucher-Maluschke J.S., Reis D.C.d., Silva S.D.B.d. (2008). The ecological engagement in the context of an Amazon river Village. Interam. J. Psychol..

[19] De Rodrigues L.R. (2020). Accessibility in the modular teaching organization system in elementary schools in riverside communities in the municipality of Abaetetuba. Braz. J. Dev..

[20] Machado F.S.N., de Carvalho M.A.P., Mataresi A., Mendonça E.T., Cardoso L.M., Yogi M.S., Rigato H.M., Salazar M. (2010). Use of telemedicine technology as a strategy to promote health care of riverside communities in the Amazon: Experience with interdisciplinary work, integrating NHS guidelines. J. Cienc. Saude Coletiva.

[21] Arrifano G.P., Alvarez-Leite J.I., Macchi B.M., Campos N.F., Augusto-Oliveira M., Santos-Sacramento L., Lopes-Araújo A., Souza-Monteiro J.R., Alburquerque-Santos R., Do Nascimento J.L.M. (2021). Living in the southern hemisphere: Metabolic syndrome and its components in Amazonian riverine populations. J. Clin. Med..

[22] Anjana R.M., Baskar V., Nair A.T.N., Jebarani S., Siddiqui M.K., Pradeepa R., Unnikrishnan R., Palmer C., Pearson E., Mohan V. (2020). Novel subgroups of type 2 diabetes and their association with microvascular outcomes in an Asian Indian population: A data-driven cluster analysis: The INSPIRED study. BMJ Open Diabetes Res..

[23] Hillesheim E., Ryan M.F., Gibney E., Roche H.M., Brennan L. (2020). Optimisation of a metabotype approach to deliver targeted dietary advice. J. Nutr..

[24] Tallman D.A., Latifi E., Kaur D., Sulaheen A., Ikizler T.A., Chinna K., Mat Daud Z.A., Karupaiah T., Khosla P. (2020). Dietary patterns and health outcomes among African American maintenance hemodialysis patients. J. Nutr..

[25] Ryan T.P. (2013). Sample Size Determination and Power.

[26] Yamane T. (1967). Statistics, an Introductory Analysis, 1967.

[27] Cabral M.M., Venticinque E.M., Rosas F.C.W. (2014). Perception of riverine people in relation to the performance and management of two distinct categories of protected areas in the Brazilian Amazon. J. Biodivers. Bras.-BioBrasil.

[28] Feio C.M.A., Fonseca F.A., Rego S.S., Feio M.N., Elias M.C., Costa E.A., Izar M.C., Paola Â.A., Carvalho A.C. (2003). Lipid profile and cardiovascular risk in Amazonians. Arq. Bras. Cardiol..

[29] Murrieta R.S.S. (2001). Dialectic of flavor: Food, ecology and daily life in riverside communities on the island of Ituqui, Baixo Amazonas, Pará. J. Rev. Antropol..

[30] Pulvers K., Scheuermann T.S., Romero D.R., Basora B., Luo X., Ahluwalia J.S. (2014). Classifying a smoker scale in adult daily and nondaily smokers. J. Nicotine Tob. Res..

[31] SBAC (2016). Brazilian Consensus for the Standardization of Laboratory Determination of Lipid Profile. Brazilian Society of Clinical Analysis. https://www.sbac.org.br/blog/2016/12/10/consenso-brasileiro-para-a-normatizacao-da-determinacao-laboratorial-do-per%EF%AC%81l-lipidico/.

[32] SBD (2017). Guidelines of the Brazilian Society of Diabetes 2017–2018.

[33] Teknomo K. K-Means Clustering Tutorials 2007. http://sigitwidiyanto.staff.gunadarma.ac.id/Downloads/files/38034/M8-NotekMeans.

[34] Mahmoud P., Beaugureau A.S.a.M. (2015). K-Means Clustering—Data Algorithms.

[35] Chatterji P., Joo H., Lahiri K. (2012). Racial/ethnic-and education-related disparities in the control of risk factors for cardiovascular disease among individuals with diabetes. Diabetes Care.

[36] Machado P.A.N., Sichieri R. (2002). Waist-hip ratio and dietary factors in adults. J. Rev. Saúde Pública.

[37] Merz C.N.B., Ramineni T., Leong D. (2018). Sex-specific risk factors for cardiovascular disease in women-making cardiovascular disease real. J. Curr. Opin. Cardiol..

[38] Khan S.U., Lone A.N., Khan M.S., Virani S.S., Blumenthal R.S., Nasir K., Miller M., Michos E.D., Ballantyne C.M., Boden W.E. (2021). Effect of omega-3 fatty acids on cardiovascular outcomes: A systematic review and meta-analysis. EClinicalMedicine.

[39] Santos-Sacramento L., Arrifano G.P., Lopes-Araújo A., Augusto-Oliveira M., Albuquerque-Santos R., Takeda P.Y., Souza-Monteiro J.R., Macchi B.M., do Nascimento J.L.M., Lima R.R. (2021). Human neurotoxicity of mercury in the Amazon: A scoping review with insights and critical considerations. J. Ecotoxicol. Environ. Saf..

[40] Basta P.C., Viana P.V.d.S., Vasconcellos A.C.S.d., Périssé A.R.S., Hofer C.B., Paiva N.S., Kempton J.W., Ciampi de Andrade D., Oliveira R.A.A.d., Achatz R.W. (2021). Mercury exposure in Munduruku indigenous communities from Brazilian Amazon: Methodological background and an overview of the principal results. Int. J. Environ. Res. Public Health.

[41] Meneses H.N.M., Oliveira-da-Costa M., Basta P.C., Morais C.G., Pereira R.J.B., De Souza S.M.S., Hacon S.S. (2022). Mercury contamination: A growing threat to riverine and urban communities in the Brazilian Amazon. Int. J. Environ. Res. Public Health.

[42] Souza-Araujo J., Andrades R., Hauser-Davis R., Lima M., Giarrizzo T. (2022). Before the Dam: A Fish-Mercury Contamination Baseline Survey at the Xingu River, Amazon Basin before the Belo Monte Dam. J. Bull. Environ. Contam. Toxicol..

[43] Cohen J. (2013). Statistical Power Analysis for the Behavioral Sciences.

[44] Fritz C.O., Morris P.E., Richler J.J. (2012). Effect size estimates: Current use, calculations, and interpretation. J. Exp. Psychol. Gen..

[45] Russell S., Norvig P. (2010). Artificial Intelligence: A Modern Approach.

[46] SBC (2016). Brazilian Society of Cardiology—7th Brazilian Guideline on Arterial Hypertension. Arq. Bras. Cardiol..

[47] WHO (2008). Waist Circumference and Waist—Hip Ratio: Report of a WHO Expert Consultation.

[48] Mariosa D.F., Ferraz R.R.N., Santos-Silva E.N. (2018). Influence of socio-environmental conditions on the prevalence of systemic arterial hypertension in two riverside communities in the Amazon, Brazil. J. Ciênc. Saúde Coletiva.

[49] Ohlsson A., Eckerdal N., Lindahl B., Hanning M., Westerling R. (2021). Non-employment and low educational level as risk factors for inequitable treatment and mortality in heart failure: A population-based cohort study of register data. J. BMC Public Health.

[50] Rarau P., Pulford J., Gouda H., Phuanukoonon S., Bullen C., Scragg R., Pham B.N., McPake B., Oldenburg B. (2019). Socio-economic status and behavioural and cardiovascular risk factors in Papua New Guinea: A cross-sectional survey. PLoS ONE.

[51] Rosengren A., Smyth A., Rangarajan S., Ramasundarahettige C., Bangdiwala S.I., AlHabib K.F., Avezum A., Boström K.B., Chifamba J., Gulec S. (2019). Socioeconomic status and risk of cardiovascular disease in 20 low-income, middle-income, and high-income countries: The Prospective Urban Rural Epidemiologic (PURE) study. Lancet Glob. Health.

[52] Fard N.A., Morales G.F., Mejova Y., Schifanella R. (2021). On the interplay between educational attainment and nutrition: A spatially-aware perspective. EPJ Data Sci..

[53] Arrighi E., Ruiz de Castilla E., Peres F., Mejía R., Sørensen K., Gunther C., Lopez R., Myers L., Quijada J., Vichnin M. (2022). Scoping health literacy in Latin America. Glob. Health Promot..

[54] De Azevedo P.L., Freitas S.R.S. (2018). Prevalence of major cardiometabolic diseases in the riverine populations from the interior of the State of Amazonas, Brazil. Acta Sci. Health Sci..

[55] Machado C.L.R., Crespo-Lopez M.E., Augusto-Oliveira M., Arrifano G.P., Macchi B.M., Lopes-Araújo A., Santos-Sacramento L., Souza-Monteiro J.R., Alvarez-Leite J.I., De Souza C.B.A. (2021). Eating in the Amazon: Nutritional status of the riverine populations and possible nudge interventions. J. Foods.

[56] Azevedo P.L. (2017). Prevalence of the Main Chronic Non-Communicable Diseases in Riverside Populations in the Interior of Amazonas. Bachelor’s thesis.

[57] Relvas A., Camargo J., Basano S., Camargo L.M.A. (2022). Prevalence of chronic noncommunicable diseases and their associated factors in adults over 39 years in riverside population in the western Brazilian amazon region. J. Hum. Growth.

[58] Omare M.O., Kibet J.K., Cherutoi J.K., Kengara F.O. (2021). A review of tobacco abuse and its epidemiological consequences. J. Public Health.

[59] Larsson S.C., Burgess S. (2022). Appraising the causal role of smoking in multiple diseases: A systematic review and meta-analysis of Mendelian randomization studies. EBioMedicine.

[60] Huerta M.C., Borgonovi F. (2010). Education, alcohol use and abuse among young adults in Britain. Soc. Sci. Med..

[61] Jefferis B., Manor O., Power C. (2008). Cognitive development in childhood and drinking behaviour over two decades in adulthood. J. Epidemiol. Community Health.

[62] Silva L.E.S., Helman B., Luz e Silva D.C., Aquino É.C., Freitas P.C., Santos R.O., Brito V.C.A., Garcia L.P., Sardinha L.M.V. (2022). Prevalence of heavy episodic drinking in the Brazilian adult population: National Health Survey 2013 and 2019. J. Epidemiol. E Serviços De Saúde.

[63] Plens J.A., Valente J.Y., Mari J.J., Ferrari G., Sanchez Z.M., Rezende L.F. (2022). Patterns of alcohol consumption in Brazilian adults. Sci. Rep..

[64] Nogueira W.P., Caetano K.A.A., Brandão G.C.G., Freire M.E.M., Reis R.K., Oliveira e Silva A.C. (2022). Harmful alcohol consumption and associated factors in riverine communities. Rev. Eletrônica De Enferm..

[65] Macinko J., Mullachery P., Silver D., Jimenez G., Neto O.L.M. (2015). Patterns of alcohol consumption and related behaviors in Brazil: Evidence from the 2013 National Health Survey (PNS 2013). PLoS ONE.

[66] Sales F.M.A.M., Silva L.M.C., Oliveira A.P.P., Reis R.C., Guerreiro J.F. (2014). Risk of excess weight/body fat and dyslipidemia associated with hemoglobin A2 levels. Rev. Para. De Med..

[67] Adams C., Murrieta R., Neves W.A. (2006). Amazonian Caboclo Societies: Modernity and Invisibility.

[68] Tomita L.Y., Cardoso M.A. (2002). Assessment of the food list and serving size of a Food Frequency Questionnaire in an adult population. Cad. De Saúde Pública.

[69] Silva A.L., Begossi A. (2009). Biodiversity, food consumption and ecological niche dimension: A study case of the riverine populations from the Rio Negro, Amazonia, Brazil. J. Environ. Dev. Sustain..

[70] Nyberg S.T., Singh-Manoux A., Pentti J., Madsen I.E., Sabia S., Alfredsson L., Bjorner J.B., Borritz M., Burr H., Goldberg M. (2020). Association of healthy lifestyle with years lived without major chronic diseases. JAMA Intern. Med..

[71] Peres M.A.C. (2011). Old age and illiteracy, a paradoxical relationship: Educational exclusion in rural contexts in the Northeast region. J. Soc. E Estado.

[72] Vasquez-Rojas W.V., Martín D., Miralles B., Recio I., Fornari T., Cano M.P. (2021). Composition of Brazil Nut (Bertholletia excels HBK), Its Beverage and By-Products: A Healthy Food and Potential Source of Ingredients. J. Foods.

[73] Matos Â.P., Matos A.C., Moecke E.H.S. (2019). Polyunsaturated fatty acids and nutritional quality of five freshwater fish species cultivated in the western region of Santa Catarina, Brazil. Braz. J. Food Technol..

[74] Arrifano G.P., Martín-Doimeadios R.C.R., Jiménez-Moreno M., Ramírez-Mateos V., da Silva N.F., Souza-Monteiro J.R., Augusto-Oliveira M., Paraense R.S., Macchi B.M., Do Nascimento J.L.M. (2018). Large-scale projects in the amazon and human exposure to mercury: The case-study of the Tucuruí Dam. J. Ecotoxicol. Environ. Saf..

[75] Hu X.F., Singh K., Chan H.M. (2018). Mercury exposure, blood pressure, and hypertension: A systematic review and dose—Response meta-analysis. J. Environ. Health Perspect..

[76] Yan J., Pan Y., Tang Z., Song Y. (2019). Mercury poisoning presenting with hypertension: Report of 2 cases. Am. J. Med..

[77] Hu X.F., Lowe M., Chan H.M. (2021). Mercury exposure, cardiovascular disease, and mortality: A systematic review and dose-response meta-analysis. J. Environ. Res..

[78] Rocha J.P.S., Lopes I.S.S., Henriques C.E.L., Minekawa T.B., Bastos M.S.C.B.O. Katuana from Baía do Guajará: Diabetes and self-reported arterial hypertension in a riverside population of Combú. Proceedings of the III Congress on Health Education in the Amazon (COESA).

[79] Rodrigues D.N., Mussi R.F.d.F., Almeida C.B.d., Nascimento Junior J.R.A., Moreira S.R., Carvalho F.O. (2020). Sociodemographic determinants associated with the level of physical activity of Bahian quilombolas, 2016 survey. J. Epidemiol. E Serviços De Saúde.

[80] Wanzeler F.S.d.C. (2017). Physical Activity and Associated Factors in Riverside Adolescents in the Amazon. Master’s Thesis.

[81] Wanzeler F.S.d.C., Nogueira J.A.D. (2019). Physical activity in rural populations of Brazil: A review of literature. Rev. Bras. De Ciência E Mov..

[82] Brasil M.S. (2008). Protocols of the Food and Nutrition Surveillance System—SISVAN in Health Care.

[83] de Araújo I.M., Antunes Paes N. (2013). Quality of anthropometric data of hypertensive users seen at the family health program and its correlation with risk factors. Texto Contexto Enferm..

[84] Pereira R.A., Sichieri R., Marins V.M. (1999). Razão cintura/quadril como preditor de hipertensão arterial. J Cad. De Saúde Pública.

[85] Rodrigues J.M.P., Da Silva G.P. (2018). The Modular Teaching Organization System (MTOS) from the perspective of graduates in the municipality of Breves—Pará. J. Rev. Bras. De Educ. Do Campo.

