# Supplementary material for: Sociobehavioral, Biological, and Health Characteristics of Riverside People in the Xingu Region, Pará, Brazil
Source: Int J Environ Res Public Health. 2023 Apr 17;20(8):5542. doi: 10.3390/ijerph20085542 (PMC10138798; doi:10.3390/ijerph20085542)
Supplement: Supplementary file 1 [file ijerph-20-05542-s001.zip › ijerph-2187814-supplementary.pdf]

Table S1. List of variables used in the construction of the clustering model by the K-means algorithm.

Variables	Description
Sociodemographic variables	- Literate or not;<br>- Education level.
Biological variables	- Sex;<br>- Age;<br>- Ethnicity;<br>- Body mass index (BMI);<br>- Waist-hip ratio (WHR);<br>- Systolic blood pressure (SBP) and Diastolic blood pressure (DBP);<br>- Blood glucose;<br>- Lipid profile: total cholesterol (TC), triglycerides (TG), high density lipoprotein (HDL), low density lipoprotein (LDL).<br>- Diseases: dyslipidemia, arterial hypertension (SAH), diabetes (DM), stroke (CVA), cardiovascular disease (CVD).
Behavioral variables	- Smoker or not;<br>- Smoking time in years and daily frequency;<br>- Consumption of alcoholic beverages and weekly frequency;<br>- Weekly frequency of physical exercises;<br>- Food consumption profile (healthy diet - including weekly consumption of fish, nuts, fruits, vegetables / bad diet - fried foods, soft drinks, sausages, among others).
